# Risk of developing gallbladder cancer in patients with gallbladder polyps detected on transabdominal ultrasound: a systematic review and meta-analysis

**DOI:** 10.1259/bjr.20220152

**Published:** 2022-07-21

**Authors:** Kieran G Foley, Zena Riddell, Bernadette Coles, S Ashley Roberts, Brian H Willis

**Affiliations:** Division of Cancer & Genetics, School of Medicine, Cardiff University, Cardiff, UK; National Imaging Academy of Wales (NIAW), Pencoed, UK; Velindre University NHS Trust Library & Knowledge Service, Cardiff, UK; Department of Clinical Radiology, University Hospital of Wales, Cardiff, UK; Institute of Applied Health Research, University of Birmingham, Birmingham, UK

## Abstract

**Objective:**

To estimate the risk of malignancy in gallbladder polyps of incremental sizes detected during transabdominal ultrasound (TAUS).

**Methods:**

We searched databases including MEDLINE, Embase, and Cochrane Library for eligible studies recording the polyp size from which gallbladder malignancy developed, confirmed following cholecystectomy, or by subsequent follow-up. Primary outcome was the risk of gallbladder cancer in patients with polyps. Secondary outcome was the effect of polyp size as a prognostic factor for cancer. Risk of bias was assessed using the Quality in Prognostic Factor Studies (QUIPS) tool. Bayesian meta-analysis estimated the median cancer risk according to polyp size. This study is registered with PROSPERO (CRD42020223629).

**Results:**

82 studies published since 1990 reported primary data for 67,837 patients. 67,774 gallbladder polyps and 889 cancers were reported. The cumulative median cancer risk of a polyp measuring 10 mm or less was 0.60% (99% credible range 0.30–1.16%). Substantial heterogeneity existed between studies (I^2^ = 99.95%, 95% credible interval 99.86–99.98%). Risk of bias was generally high and overall confidence in evidence was low. 13 studies (15.6%) were graded with very low certainty, 56 studies (68.3%) with low certainty, and 13 studies (15.6%) with moderate certainty. In studies considered moderate quality, TAUS monitoring detected 4.6 cancers per 10,000 patients with polyps less than 10 mm.

**Conclusion:**

Malignant risk in gallbladder polyps is low, particularly in polyps less than 10 mm, however the data are heterogenous and generally low quality. International guidelines, which have not previously modelled size data, should be informed by these findings.

**Advances in knowledge:**

This large systematic review and meta-analysis has shown that the mean cumulative risk of small gallbladder polyps is low, but heterogeneity and missing data in larger polyp sizes (>10 mm) means the risk is uncertain and may be higher than estimated.

Studies considered to have better methodological quality suggest that previous estimates of risk are likely to be inflated.

## Introduction

Gallbladder polyps are commonly detected in adults during transabdominal ultrasound examination (TAUS).^1^ Gallbladder polyps can be separated into two categories; true polyps, or adenomas, that have malignant potential, and pseudopolyps consisting predominately of cholesterol, which have no malignant potential at all. The latter group is estimated to constitute 70% of all reported gallbladder polyps.^2^

Gallbladder cancer has been shown to develop from polypoid adenomas.^3,4^ More than 200,000 patients are diagnosed with gallbladder cancer each year worldwide.^5^ Gallbladder cancer carries a poor prognosis (15–20% 5-year survival) because patients commonly present at an advanced stage of disease and are unsuitable for radical therapy.^6^ The risk of malignant transformation of polyps to cancer is thought to be small, however accurate estimates of risk are unknown. Predicting which of the many patients with gallbladder polyps will develop gallbladder cancer is extremely difficult, but clinically important.

The assessment and monitoring of gallbladder polyps represent an ongoing clinical challenge that requires considerable resources from radiology departments around the world. Several international societies have attempted to provide evidence-based clinical guidance, based on size thresholds for intervention. Generally, it is recommended that patients with gallbladder polyps measuring 10 mm or more should undergo cholecystectomy. Recently updated European guidelines^7^ recommend ultrasound monitoring for up to 2 years in patients with polyps measuring 6 mm or more, provided polyp size is stable, or for polyps 5 mm or less if risk factors are present. In contrast, the Canadian Association of Radiologists recently endorsed the American College of Radiology recommendations that surveillance of polyps measuring 7 mm or more should be performed for up to 2 years, with polyps less than 7 mm not requiring follow-up.^8^ The available evidence is largely considered to be low quality,^1,2,9–11^ and international guidance has never modelled polyp size for malignant risk to justify their recommendations for appropriate intervention. Additional limitations include strong selection, detection, and reporting bias which significantly hinders confidence in any current estimated malignant risk.

Therefore, to address this gap, a systematic review and meta-analysis was conducted to establish the overall risk of gallbladder cancer in patients with polyps detected by TAUS. We examined TAUS measured polyp size as a prognostic factor for gallbladder cancer and explored other potentially important clinical co-variates for their associated malignant risk.

## Methods and materials

This study was prospectively registered with PROSPERO (CRD42020223629) and results were reported following the Preferred Reporting Items for Systematic Reviews and Meta-analysis (PRISMA) guidelines.^12^

### Search strategy

A comprehensive search strategy using Medical Subject Headings (MeSH) and free-text terms was designed for this systematic review using MEDLINE. This strategy was adapted to run in the following electronic databases: MEDLINE, Embase, Cochrane Library, Cumulative Index of Nursing and Allied Health Literature (CINAHL), Scopus, Web of Science, and ClinicalTrials.gov. (Supplementary Material 1) The initial search was performed on October 28, 2020, and updated on December 4, 2020. The search was limited to English language.

### Study selection

The systematic review included randomised control trials, observational cohort, cross-sectional and case–control studies published since 1990. We included studies that reported consecutive or random primary data in adult participants (18 years or older), diagnosed with a gallbladder polyp on TAUS, that recorded the size of polyp from which a gallbladder malignancy occurred, confirmed either following cholecystectomy, or by monitoring the polyp to determine its natural history. A monitoring period of at least 12 months was required. A polyp is often termed a mass once it measures 30 mm, however, to maximise the capture of continuous data, sizes of polypoid lesions more than 30 mm were also recorded. Studies were excluded that did not contain any primary data or did not provide polyp or cancer measurements. Attempt was made to discover translations of any non-English language article that was inadvertently retrieved. Reference lists of all eligible studies were checked and underwent citation tracking for additional eligible studies. Search of the grey literature was not performed.

### Outcomes

The pre-specified primary outcome was the risk of gallbladder cancer in adult patients with polyps detected by TAUS. The secondary outcome was the effect of polyp size as a prognostic factor for gallbladder cancer. Additional secondary outcomes were the malignant risk of associated clinical co-variates: age at diagnosis, gender, presence of gallstones, presence of symptoms, and the presence of single or multiple polyps.

### Data extraction

Two investigators (KGF/ZR) independently screened all titles and abstracts, assessed full texts for eligibility, and extracted data based on the CHARMS^13^ and CHARMS-PF^14^ checklists. Disagreements were resolved after review by a third investigator (SAR). Data extracted (Supplementary Material 1) included study identifiers, study design, setting and population characteristics, sample size, polyp and cancer size, and follow-up. Where an included study reported missing data, the corresponding author was contacted inviting them to share the complete data set.

### Quality assessment

Risk of bias was assessed using the Quality in Prognostic Factor Studies (QUIPS) tool for each study.^15^ The strength of the overall weight of evidence for both primary and secondary outcomes was judged using the Grading of Recommendations Assessment, Development and Evaluation (GRADE) working group methodology.^16^ (Supplementary Material 1)

### Data analysis

A Bayesian meta-analysis model which incorporated the effects of polyp size and other covariates on the risk of cancer was developed. This was a random intercept and random gradient model to allow the effects of polyp size on the risk of cancer to vary across studies. The model was expanded to account for extensive missing data amongst the response and the predictor variables. The data were modelled by assuming separate multinomial distributions for the number of cancers and polyps at different sizes and imputing new data for each iteration of the Bayesian model. As a result, all eligible studies could be included in the analysis. The model was supplemented with individual patient data, where available. The Bayesian meta-analysis model^17,18^ was developed in JAGS^19^ interfacing with R^20^ via the rjags package.^21^ (Supplementary Material 1) Between-study heterogeneity was assessed by inspection of prediction plots, and the I^2^ statistic.^22,23^ To assess the effects of the GRADE rating on the Bayesian model, sensitivity analyses were conducted where studies rated with very low certainty were first excluded, followed by the exclusion of low and very low certainty studies.

## Results

**Figure 1.**
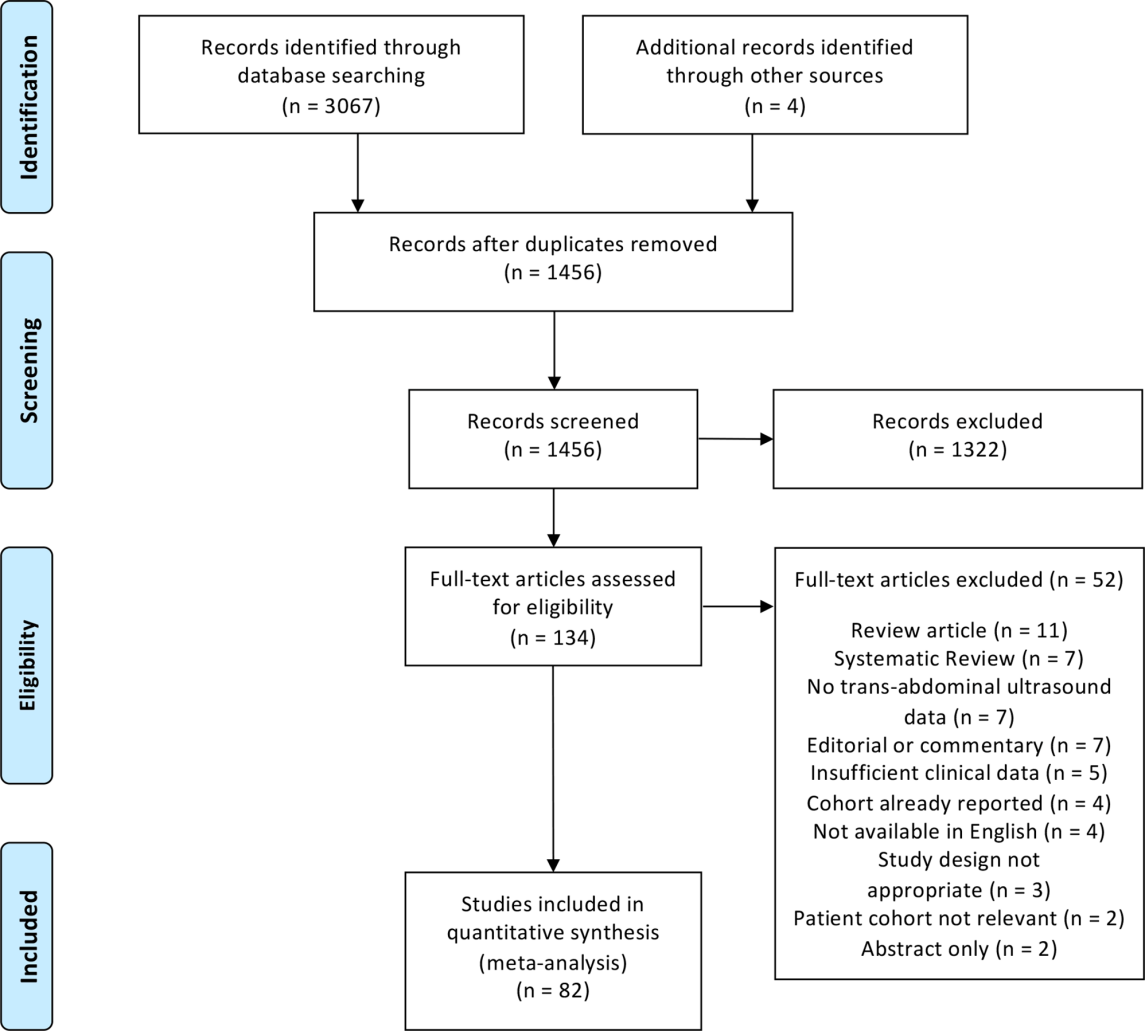
Study selection process.

The initial search identified 3067 studies, of which 1615 were duplicates. Four additional studies were identified through other sources. The titles and abstracts of 1456 studies were screened and after screening, 1322 records were excluded for being irrelevant to this systematic review, leaving 134 full-text articles for review. Both reviewers identified 122 of the 134 full-text articles (91.0%) and the remaining 12 were included after agreement by the third reviewer. Of the 134 full-text articles, 52 were excluded (agreed by both reviewers) leaving 82 articles^24–105^ published since 1990 for inclusion (Figure 1). Important characteristics of the 82 included studies are detailed in Table 1.

**Table 1.** Studies reporting transabdominal ultrasound measurements of gallbladder polyps and malignancies that met the inclusion criteria

Author	Year	Country	Design	Sites	Start date	End date	Patients	Median age (months)	Female	Polyps	Cancers	Malignancy rate	Cholecystectomy	Monitoring	Median follow-up (months)
Abdullah et al^24^	2019	UK	Retrospective	1	2011	2013	244	NR *	160 (65.6%)	201	2	1.0%	43 (21.4%)	137 (68.2%)	36
Ahmed et al^25^	2013	UK	Retrospective	1	2005	2010	39	51.4	29 (22.1%)	39	0	0.0%	39 (100.0%)	0 (0.0%)	NR
Akyurek et al^26^	2005	Turkey	Retrospective	1	2000	2004	56	48	16 (28.6%)	56	0	0.0%	56 (100.0%)	0 (0.0%)	NR
Al Manasra et al^27^	2018	Jordan	Retrospective	1	2002	2016	46	54	31 (67.4%)	46	5	7.7%	46 (100.0%)	0 (0.0%)	NR
Aldouri et al^28^	2009	UK	Retrospective	1	1998	2006	2429	58	NR	2429	28	10.9%	2429 (100.0%)	0 (0.0%)	NR
Aliyazicioglu et al^29^	2017	Turkey	Retrospective	1	2004	2015	185	44.6	94 (50.8%)	185	2	1.1%	185 (100.0%)	0 (0.0%)	NR
Ansari et al^30^	2007	Bangladesh	Prospective	1	2002	2004	57	NR	NR	57	1	1.8%	37 (64.9%)	26 (45.6%)	18
Azuma et al^31^	2001	Japan	Retrospective	1	1989	1998	89	NR	NR	89	24	27.0%	89 (100.0%)	0 (0.0%)	NR
Cairns et al^32^	2012	UK	Retrospective	1	2000	2011	986	57.1	541 (54.9%)	986	1	0.1%	134 (13.6%)	467 (47.4%)	39.3
Cha et al^33^	2011	South Korea	Retrospective	1	2003	2009	210	NR	101 (48.1%)	210	65	31.0%	210 (100.0%)	0 (0.0%)	NR
Channa et al^34^	2009	Pakistan	Retrospective	1	1999	2008	28	47.5	3 (10.7%)	59	3	1.2%	28 (47.5%)	0 (0.0%)	NR
Chattopadhyay et al ^35^	2005	UK	Retrospective	1	1993	2002	23	56.8	16 (69.6%)	23	3	13.0%	23 (100.0%)	0 (0.0%)	NR
Cheon et al ^36^	2009	South Korea	Retrospective	1	1996	2006	94	50	NR	94	4	4.3%	94 (100.0%)	0 (0.0%)	NR
Chijiiwa et al ^37^	1994	Japan	Retrospective	1	1982	1990	44	NR	24 (54.5%)	44	12	27.3%	44 (100.0%)	0 (0.0%)	NR
Choi et al ^38^	2008	South Korea	Retrospective	1	2006	2007	59	NR	16 (27.1%)	262	3	5.1%	59 (22.5%)	0 (0.0%)	NR
Chou et al ^39^	2017	Taiwan	Retrospective	1	2004	2013	1204	51.8	527 (43.8%)	1204	39	3.2%	194 (16.1%)	1010 (83.9%)	72
Colecchia et al ^40^	2009	Italy	Prospective	1	1999	2001	56	48.3	22 (39.3%)	56	0	0.0%	0 (0.0%)	53 (94.6%)	60
Collett et al ^41^	1998	New Zealand	Prospective	1	1989	1994	38	56	NR	38	0	0.0%	0 (0.0%)	22 (57.9%)	60
Corwin et al ^42^	2011	USA	Retrospective	1	1999	2001	346	51.6	NR	346	0	0.0%	42 (12.1%)	346 (100.0%)	96
Csendes et al ^43^	2001	Chile	Prospective	1	1987	1996	111	47	60 (54.1%)	111	0	0.0%	27 (24.3%)	98 (88.3%)	71
Dacka et al ^44^	2004	Poland	Retrospective	1	1998	2002	25	NR	14 (56.0%)	25	0	0.0%	25 (100.0%)	0 (0.0%)	NR
Damore et al ^45^	2001	USA	Retrospective	1	1988	1995	41	47.4	18 (43.9%)	41	0	0.0%	41 (100.0%)	0 (0.0%)	NR
Donald et al ^46^	2013	USA	Retrospective	1	2002	2011	27	NR	5 (18.5%)	27	3	11.1%	18 (66.7%)	0 (0.0%)	NR
Drews et al ^47^	2005	Poland	Retrospective	1	1993	2003	39	NR	17 (43.6%)	39	1	2.6%	39 (100.0%)	0 (0.0%)	NR
Escalona et al ^48^	2006	Chile	Retrospective	1	1991	2004	123	NR	85 (69.1%)	123	1	0.8%	123 (100.0%)	0 (0.0%)	NR
French et al ^49^	2013	Canada	Retrospective	1	2000	2010	262	49.7	184 (70.2%)	50	0	1.1%	262 (100.0%)	14 (5.3%)	NR
Fujiwara et al ^50^	2020	Japan	Retrospective	1	2003	2019	227	NR	99 (43.6%)	227	23	10.1%	227 (100.0%)	227 (100.0%)	60
Guo et al ^51^	2015	China	Retrospective	1	1999	2012	160	NR	90 (56.3%)	160	14	8.8%	160 (100.0%)	0 (0.0%)	NR
Heitz et al ^52^	2019	Germany	Prospective	Multi	2002	2013	50	57.8	NR	153	6	0.0%	0 (0.0%)	16 (32.0%)	132
Huang et al ^53^	2001	Taiwan	Retrospective	1	1990	1998	153	NR	76 (49.7%)	62	9	3.9%	153 (100.0%)	0 (0.0%)	NR
Isozaki et al ^54^	1995	Japan	Retrospective	1	1978	1992	62	NR	31 (50.0%)	144	29	14.5%	62 (43.1%)	0 (0.0%)	NR
Ito et al ^55^	2009	USA	Retrospective	1	1996	2007	417	NR	229 (54.9%)	417	1	0.2%	80 (19.2%)	143 (34.3%)	17
Jang et al ^56^	2009	South Korea	Prospective	1	2006	2007	144	57.6	72 (50.0%)	126	8	20.1%	144 (100.0%)	0 (0.0%)	NR
Jeong et al ^57^	2020	South Korea	Retrospective	1	2006	2017	535	NR	300 (56.1%)	535	84	15.7%	535 (100.0%)	0 (0.0%)	NR
Kamali Polat et al ^58^	2010	Turkey	Retrospective	1	Missing	Missing	34	47.2	14 (41.2%)	34	1	2.9%	31 (91.2%)	0 (0.0%)	NR
Khan et al ^59^	2012	Saudi Arabia	Retrospective	1	2008	2012	26	40	19 (73.1%)	26	1	3.8%	26 (100.0%)	0 (0.0%)	NR
Kim et al ^60^	2016	South Korea	Retrospective	1	2007	2011	53	NR	27 (50.9%)	53	8	15.1%	35 (66.0%)	18 (34.0%)	46.4
Konstantinidis et al ^61^	2012	USA	Retrospective	1	2000	2010	213	52	147 (69.0%)	213	6	2.8%	213 (100.0%)	20 (9.4%)	15.5
Koundouris et al ^62^	2001	Greece	Retrospective	1	1994	2000	35	52	21 (60.0%)	35	7	20.0%	35 (100.0%)	0 (0.0%)	NR
Kratzer et al ^63^	2008	Germany	Prospective	1	1996	1996	31	NR	8 (25.8%)	31	0	0.0%	0 (0.0%)	22 (71.0%)	84
Kubota et al ^64^	1995	Japan	Retrospective	1	1978	1994	72	NR	32 (44.4%)	72	16	22.2%	72 (100.0%)	12 (16.7%)	12
Kwon et al ^65^	2009	South Korea	Retrospective	1	1992	2005	291	NR	151 (51.9%)	291	35	12.0%	291 (100.0%)	0 (0.0%)	NR
Lee et al ^66^	2016	South Korea	Retrospective	1	2002	2016	126	NR	66 (52.4%)	516	24	6.3%	126 (24.4%)	0 (0.0%)	NR
Lee et al ^67^	2019	South Korea	Retrospective	1	2005	2014	516	NR	219 (42.4%)	109	1	4.7%	516 (100.0%)	109 (21.1%)	60
Liu ^68^	2018	China	Retrospective	1	2013	2017	109	NR	60 (55.0%)	109	23	21.1%	109 (100.0%)	0 (0.0%)	NR
Maciejewski et al ^69^	2014	Poland	Retrospective	1	2010	2013	64	52.9	NR	64	1	1.6%	64 (100.0%)	0 (0.0%)	NR
Mainprize et al ^70^	2000	UK	Retrospective	1	1993	1997	38	NR	19 (50.0%)	18	2	11.1%	34 (89.5%)	0 (0.0%)	NR
Matlok et al ^71^	2013	Poland	Retrospective	1	1997	2012	152	NR	94 (61.8%)	152	1	0.7%	152 (100.0%)	8 (5.3%)	NR
Matos et al ^72^	2010	Portugal	Retrospective	1	2003	2007	93	NR	62 (66.7%)	93	2	2.2%	86 (92.5%)	0 (0.0%)	NR
Metman et al ^73^	2020	Netherlands	Retrospective	2	2010	2010	108	56	63 (58.3%)	108	0	0.0%	108 (100.0%)	35 (32.4%)	NR
Moriguchi et al ^74^	1996	Japan	Prospective	1	1988	1988	109	54	58 (53.2%)	28	1	0.9%	0 (0.0%)	109 (100.0%)	37.2
Okamoto et al ^75^	1999	Japan	Retrospective	1	1986	1993	1,0926	NR	NR	1,0926	19	0.2%	33 (0.3%)	0 (0.0%)	NR
Onda et al ^76^	2020	Japan	Retrospective	1	2009	2014	139	NR	55 (39.6%)	139	16	11.5%	139 (100.0%)	80 (57.6%)	NR
Ostapenko et al ^77^	2020	USA	Retrospective	1	2014	2019	98	NR	NR	98	0	0.0%	98 (100.0%)	0 (0.0%)	NR
Park et al ^78^	2008	South Korea	Retrospective	1	1988	2006	689	NR	542 (78.7%)	689	25	3.6%	180 (26.1%)	689 (100.0%)	60
Park et al ^79^	2009	South Korea	Retrospective	1	1995	2005	1558	NR	723 (46.4%)	1558	34	3.6%	0 (0.0%)	1558 (100.0%)	37.2
Park et al ^80^	2015	South Korea	Retrospective	1	1997	2012	836	47	387 (46.3%)	836	56	6.7%	836 (100.0%)	184 (22.0%)	NR
Patel et al ^81^	2019	UK	Retrospective	1	2008	2013	558	52	297 (53.2%)	558	3	0.5%	89 (15.9%)	168 (30.1%)	23.5
Pedersen et al ^82^	2012	Denmark	Retrospective	1	2008	2009	203	54	114 (56.2%)	203	0	0.0%	13 (6.4%)	31 (15.3%)	24
Pickering et al ^83^	2020	Ireland	Retrospective	4	2015	2018	134	53	78 (58.2%)	134	6	4.5%	134 (100.0%)	0 (0.0%)	NR
Rafaelsen et al ^84^	2020	Denmark	Prospective	1	2007	2009	154	62	100 (64.9%)	154	0	0.0%	0 (0.0%)	154 (100.0%)	120
Sahiner et al ^85^	2018	Turkey	Retrospective	1	2008	2013	159	NR	NR	159	8	5.0%	96 (60.4%)	0 (0.0%)	NR
Sarici et al ^86^	2017	Turkey	Retrospective	1	2005	2015	109	45	69 (63.3%)	109	15	2.2%	109 (100.0%)	60 (55.0%)	22.2
Sarkut et al ^87^	2013	Turkey	Retrospective	1	1996	2012	138	55	91 (65.9%)	138	21	15.2%	138 (100.0%)	0 (0.0%)	NR
Shah ^88^	2010	Nepal	Retrospective	1	2004	2009	32	40	23 (74.2%)	32	2	6.3%	32 (100.0%)	0 (0.0%)	NR
Shin et al ^89^	2009	South Korea	Retrospective	1	1994	2007	145	48	60 (41.4%)	145	8	5.5%	145 (100.0%)	91 (62.8%)	NR
Shinkai et al ^90^	1998	Japan	Retrospective	1	1990	1995	60	NR	25 (41.7%)	60	1	13.8%	19 (31.7%)	0 (0.0%)	NR
Spaziani et al ^91^	2019	Italy	Retrospective	1	2005	2018	38	53	23 (60.5%)	38	10	26.3%	38 (100.0%)	0 (0.0%)	NR
Sugiyama et al ^92^	2000	Japan	Retrospective	1	1988	1997	194	52	105 (54.1%)	194	11	5.7%	58 (29.9%)	125 (64.4%)	31.2
Sun et al ^93^	2004	China	Retrospective	1	1994	2002	194	45.7	101 (52.1%)	194	11	1.7%	194 (100.0%)	0 (0.0%)	NR
Sun et al ^94^	2019	China	Retrospective	1	2003	2016	686	NR	383 (55.8%)	686	10	1.5%	686 (100.0%)	686 (100.0%)	24
Sung et al ^95^	2014	South Korea	Retrospective	1	2009	2011	228	51.6	133 (58.3%)	253	18	5.7%	253 (100.0%)	0 (0.0%)	NR
Szpakowski et al ^96^	2020	USA	Retrospective	Multi	1995	2014	3,5856	50	1,8645 (52.0%)	3,5856	19	0.05%	5731 (16.0%)	3,5856 (100.0%)	NR
Terzi et al ^97^	2000	Turkey	Retrospective	1	1988	1998	100	NR	74 (74.0%)	100	26	26.0%	100 (100.0%)	0 (0.0%)	NR
Terzioglu et al ^98^	2017	Turkey	Retrospective	1	2010	2016	278	NR	187 (67.3%)	278	8	7.1%	278 (100.0%)	0 (0.0%)	NR
Ungarreevittaya et al ^99^	2018	Thailand	Retrospective	1	2017	2017	85	NR	47 (55.3%)	85	5	2.9%	85 (100.0%)	0 (0.0%)	NR
Velidedeoglu et al ^100^	2017	Turkey	Retrospective	1	2000	2012	82	48.1	47 (57.3%)	82	0	0.0%	82 (100.0%)	0 (0.0%)	NR
Wu et al ^101^	2019	China	Retrospective	1	2011	2017	1561	49.5	925 (59.3%)	1561	3	5.9%	1561 (100.0%)	0 (0.0%)	NR
Xu et al ^102^	2017	China	Retrospective	1	2008	2015	1468	NR	743 (50.6%)	1468	24	0.2%	1446 (98.5%)	0 (0.0%)	NR
Yang et al ^103^	1992	China	Retrospective	1	1982	1990	172	44.3	79 (45.9%)	172	13	7.6%	172 (100.0%)	0 (0.0%)	NR
Yeh ^104^	2001	Taiwan	Retrospective	1	1991	1999	123	NR	69 (56.1%)	123	7	1.6%	123 (100.0%)	0 (0.0%)	NR
Zielinski et al ^105^	2009	USA	Retrospective	1	1996	2007	130	NR	85 (65.4%)	130	10	7.7%	130 (100.0%)	25 (19.2%)	32

* NR not reported. Total percentages of patients treated with cholecystectomy and monitoring may not add up to 100% (total can include patients followed-up before or after cholecystectomy and patients lost to follow-up).

The 52 excluded articles were either review articles^106–116^ or systematic reviews,^2,9,10,117–120^ contained no TAUS data,^121–127^ were editorials, commentaries or reports,^128–134^ contained insufficient clinical data,^3,135–138^ contained patient cohorts previously reported,^139–142^ were not available in English,^143–146^ had study design not relevant for this review,^147–149^ included a patient cohort not relevant to this review,^150,151^ or were abstracts only.^152,153^

Overall, 67,837 patients were included for evidence synthesis. In total, 67,774 gallbladder polyps and 889 gallbladder cancers were reported. The median age ranged between 40 and 62, and 57,670 were male (73.7%). All patients had gallbladder polyps detected by TAUS. In total, 20,543 were evaluated following cholecystectomy. More than half of all polyps (*n* = 41,041, 53.1%) were monitored with TAUS to determine their natural history. The two largest studies^75,96^ provided 46,782 patients, but only 38 cancers.

There were 82 studies which provided data on the number of gallbladder polyps and cancers.^24–105^ Sixty studies provided data on at least one polyp size and the associated number of gallbladder cancers that developed in polyp sizes up to 15 mm.^24–26,29–33,35–37,39–48,50,51,55,57–65,68–73,75–78,80–85,87–89,91,92,94,96,97,100,103,105^ Size measurements could be extracted in 59,225 polyps and 425 malignant polyps, respectively, from these studies. In one study, the authors provided individual patient data on 558 patients.^81^

16 studies (19.5%) reported cohorts with zero cancer events within the first year of follow-up.^25,26,36,40–45,49,63,73,77,82,84,100^ 44 studies reported non-zero cancer events in one or more polyp sizes.^24,29–33,36,37,39,50,61,64,65,68,76,78,80,81,85,88,91,92,96,97,103,105^ 10 studies reported on the number of cancers less than 20 mm, but not the number of polyps.^36,37,39,46,47,54,75,78,88,94^

**Figure 2.**
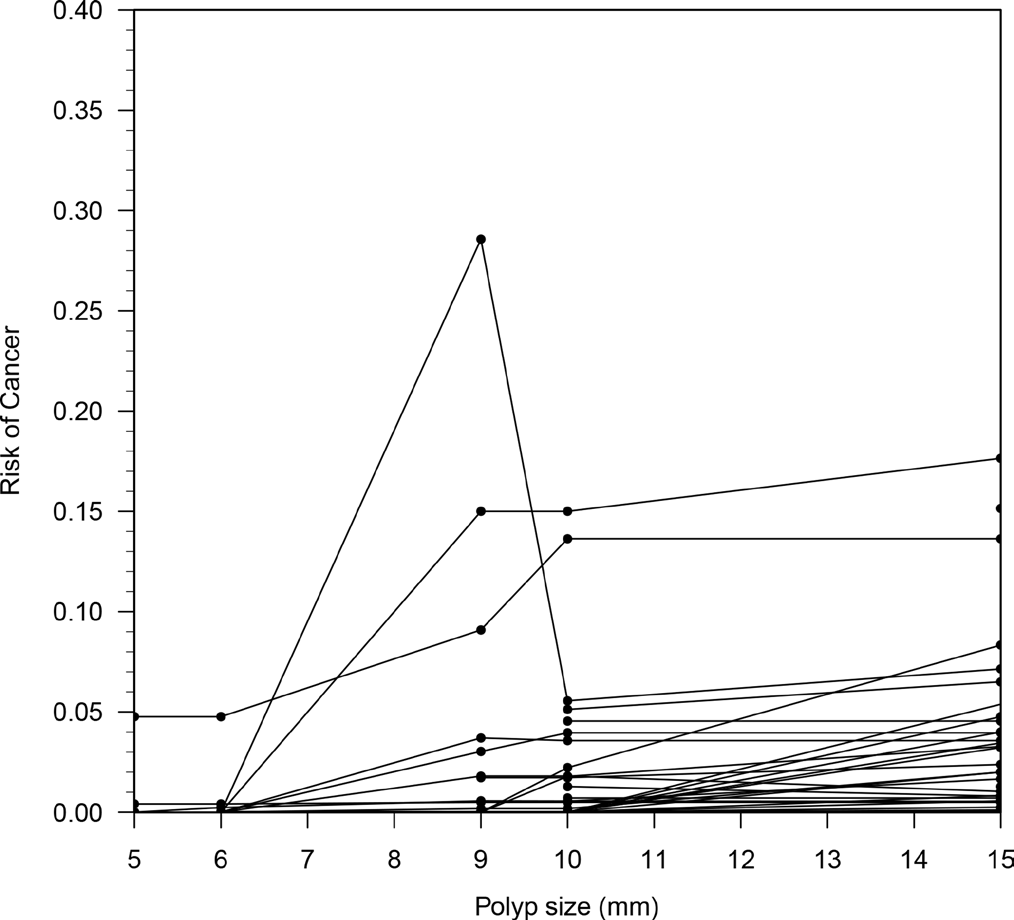
Distribution of cancer risk according to gallbladder polyp size measured by transabdominal ultrasound across all included studies. Each dot represents the cancer risk at a particular polyp size for a single study. Studies which reported cancer risk at multiple polyp sizes are depicted by the line connecting the dots associated with the study. The majority of studies showed the risk of cancer to be less than 0.1 for polyp sizes up to 15 mm.

Substantial heterogeneity was measured between studies (I^2^ = 99.95%, 95% credible interval 99.86–99.98%). The distribution of included studies at different size thresholds is shown in Figure 2 and demonstrates the heterogeneity across studies, although most studies were concentrated in a region with a probability of cancer of less than 0.03. Data reported at subsequent time points were limited, so malignant risk over time could not be determined.

**Figure 3.**
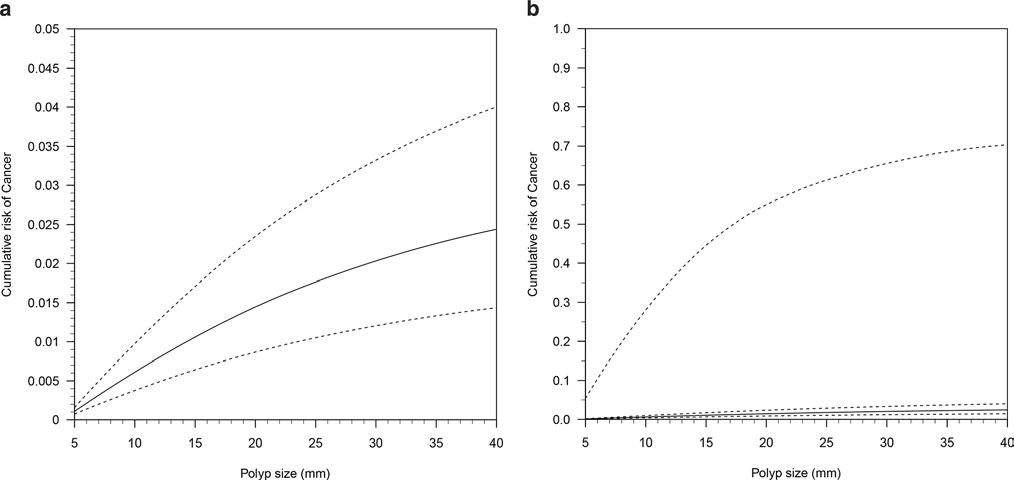
(**a**) Meta-analysis summary model showing cumulative risk of gallbladder cancer as a function of polyp size and associated 95% credible interval limits (dashed lines). (**b**) 95% prediction regions for the estimated cumulative risk. The prediction region covers nearly all the probability space for high thresholds suggesting that the heterogeneity and missing data introduces substantial uncertainty to the model. The summary mean curve and 95% credible region are included but are close to the x-axis. The upper boundary (dashed) is readily apparent, and the lower boundary of the 95% credible region is the dashed line closest to the x-axis.

A Bayesian meta-analysis model was developed to accommodate substantial missing data across the studies. As a result, it was possible to include all 82 studies in the analysis. The model demonstrated an increased risk of cancer as polyp size increased (Figure 3a). For example, a mean polyp size of 13.9 mm had a mean risk of 1 in 100. However, there was considerable uncertainty with this estimate due to study heterogeneity and this uncertainty increased with threshold size, illustrated by the widening credible ranges, which may be explained by increased missing data at higher polyp sizes. Figure 3b shows the 95% prediction region for the predicted risk from the model. This demonstrates the effects of between-study heterogeneity on the uncertainty of the risk estimates. The prediction region is wide and increases with polyp size to around 60% suggesting substantial uncertainty in the model estimates. The addition of associated co-variates (age, gender, presence of gallstones, symptoms, and single or multiple polyps) to the model did not substantially change the Deviance Information Criterion (DIC) of the Bayesian model and therefore were excluded (Supplementary Material 1).

The median cancer risk of polyps measuring 5 mm and 10 mm across all studies was 0.14% (99% credible range 0.08–0.26%) and 0.60% (0.30–1.16%), respectively. Thus, the number of patients with polyps measuring 5 mm and 10 mm or less needed to detect one cancer is 714.3 and 166.7, respectively, equating to 13.2 and 64.4 cancers per 10,000 patients. The point estimates and cumulative cancer risk with 99% credible intervals for incremental polyp size is provided in Table 2. A probability matrix, showing incremental sizes of polyps with corresponding cancer risk, is included in Supplementary Material 1.

**Table 2.** Point estimate and cumulative cancer risk for incremental polyp size with 99% credible intervals

Polyp size	Median risk	Polyp size	Median risk
5 mm	0.14% (0.08–0.26%%)	23 mm	1.64% (0.79–3.25%)
6 mm	0.22% (0.12–0.42%%)	24 mm	1.70% (0.81–3.37%)
7 mm	0.31% (0.16–0.59%)	25 mm	1.76% (0.84–3.49%)
8 mm	0.41% (0.21–0.78%)	26 mm	1.82% (0.87–3.6%)
9 mm	0.51% (0.26–0.97%)	27 mm	1.87% (0.89–3.71%)
10 mm	0.60% (0.30–1.16%)	28 mm	1.92% (0.91–3.81%)
11 mm	0.70% (0.35–1.36%)	29 mm	1.97% (0.94–3.91%)
12 mm	0.80% (0.39–1.54%)	30 mm	2.02% (0.96–4.01%)
13 mm	0.89% (0.44–1.73%)	31 mm	2.07% (0.98–4.11%)
14 mm	0.98% (0.48–1.91%)	32 mm	2.11% (1.00–4.20%)
15 mm	1.06% (0.52–2.08%)	33 mm	2.16% (1.02–4.29%)
16 mm	1.14% (0.56–2.25%)	34 mm	2.20% (1.04–4.38%)
17 mm	1.22% (0.59–2.41%)	35 mm	2.24% (1.06–4.46%)
18 mm	1.30% (0.63–2.56%)	36 mm	2.28% (1.08–4.54%)
19 mm	1.37% (0.66–2.71%)	37 mm	2.32% (1.09–4.62%)
20 mm	1.44% (0.70–2.85%)	38 mm	2.36% (1.11–4.69%)
21 mm	1.51% (0.73–2.99%)	39 mm	2.39% (1.13–4.77%)
22 mm	1.58% (0.76–3.12%)	40 mm	2.43% (1.14–4.84%)

### Risk of bias assessment

The majority of studies (*n* = 68, 82.9%) were assessed as having high risk of bias due to their observational nature, and the remaining 14 (17.1%) as moderate risk of bias (Supplementary Material 1). According to the GRADE working group methodology,^16^ 13 studies (15.6%) were graded with very low certainty, 56 studies (68.3%) with low certainty, and 13 studies (15.6%) with moderate certainty (Supplementary Material 1). The overall confidence in the result of the quantitative synthesis was summarised as low.

### Sensitivity analysis

**Figure 4.**
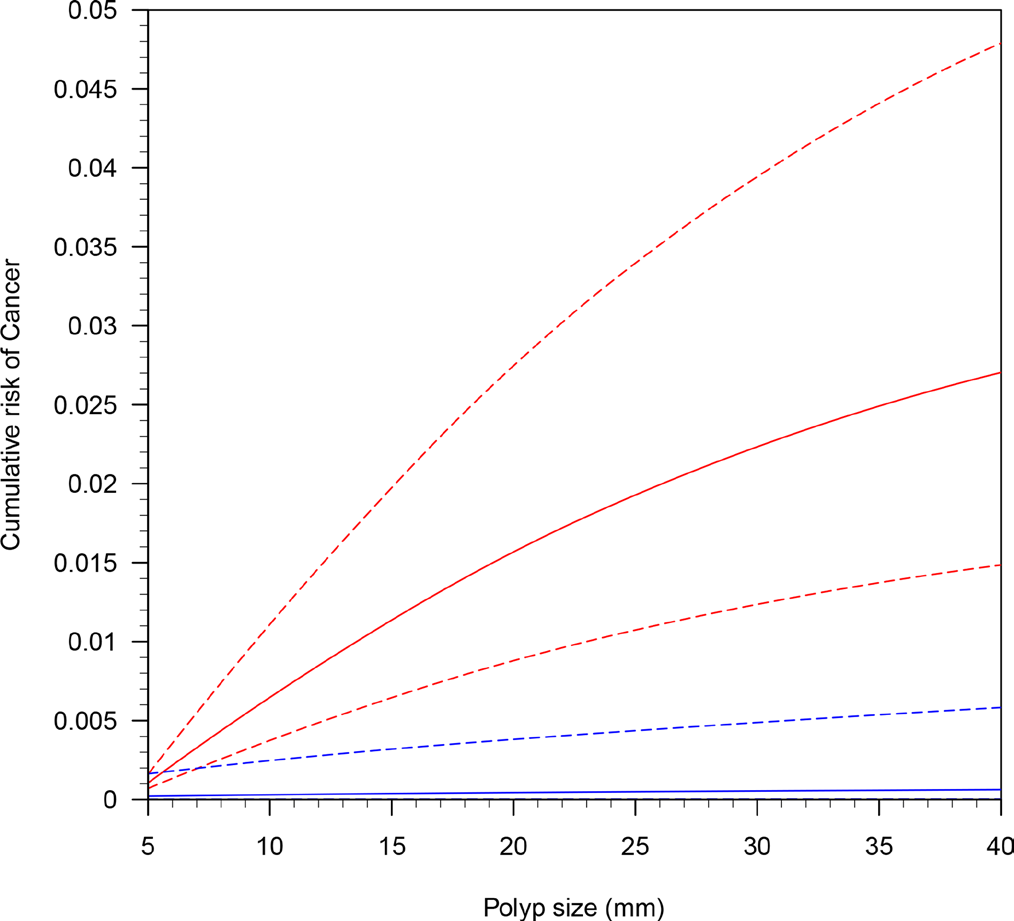
Sensitivity analysis of cumulative risk of cancer with credible intervals related to study quality. Studies rated low certainty and above (69 studies; 66,985 patients, 870 cancers) are red. Studies rated moderate certainty and above (13 studies, 51,442 patients, 100 cancers) are blue.

The effect of methodological quality on the median cancer risk was tested in sensitivity analysis (Figure 4). Compared with the overall median curve, excluding studies with a very low certainty rating had little effect on the estimated risk. However, confining the analyses to those studies with moderate certainty or higher (13 studies) substantially lowered the median risk curve. This is due to the two largest studies, which reported only 38 cancers in 46,782 patients (0.08%), having substantially lower cancer rates than the other studies in the meta-analysis.

In studies considered moderate quality, the median cancer risk of polyps measuring 5 mm and 10 mm or less reduced considerably to 0.03 and 0.04%, respectively. This increased the number of patients needed to detect one cancer to 2754.8 and 2167.8, equating to 3.6 and 4.6 cancers per 10,000 patients with polyps measuring 5 mm and 10 mm or less, respectively.

## Discussion

This systematic review and meta-analysis of more than 67,000 patients is the first comprehensive meta-analysis to model the risk of malignancy in gallbladder polyps. The study has shown that the estimated risk of malignancy in patients with gallbladder polyps is lower than previously reported and is extremely low in polyps measuring less than 10 mm.

Presently, studies are mostly low quality which affects the estimates of malignant risk presented in this meta-analysis, however the risk of cancer reported in the two largest and higher quality studies^75,96^ was far lower than the remainder of small, low-quality studies, which were likely to report inflated risk. The findings of this meta-analysis suggest that the risk of malignancy in gallbladder polyps is very low, suggesting that the monitoring of gallbladder polyps, particularly small polyps, may not be clinically or cost-effective in some healthcare systems. However, given the uncertainty introduced by the low quality studies, the clinical and cost effectiveness of monitoring small polyps requires further investigation.

Previous work has attempted to estimate the risk of malignancy in ultrasound detected gallbladder polyps. A large recent study hypothesised that the true risk of gallbladder polyps may not be as great as previously reported. A retrospective study reported outcomes of gallbladder polyps over a 20-year period in a population of more than 600,000.^96^ The unadjusted gallbladder cancer rate per 100,000 person-years was 11.3 (95% confidence intervals 6.2–16.3) and increased with greater polyp size, from 1.3 (95% confidence intervals 0.7–6.5) in polyps less than 6 mm to 128.2 (95% confidence intervals 9.4–217.0) in polyps 10 mm or greater. Additionally, gallbladder cancer rates in this cohort study were similar in patients with and without polyps on initial TAUS (0.053% *vs*  0.054%, respectively). These data were collected retrospectively, and the proportion of pseudopolyps was not reported. The study demonstrated the apparent benign natural history and slow growth of most polyps, but firm estimates of median cancer risk cannot be extrapolated from this study due to its limitations.

Further, we have confirmed that increasing polyp size is an important prognostic factor for the development of malignancy, but an optimal size threshold for intervention remains uncertain. Gallbladder polyp size is commonly reported at TAUS because the reliability and reproducibility of size measurements is excellent.^154^ The decision to intervene in patients with gallbladder polyps is contentious, but important, as many patients undergo cholecystectomy every year for gallbladder polyps. An arbitrary threshold of 10 mm is commonly cited for intervention in the literature,^39,48,65,67,79,80,86,89,102^ though larger size thresholds have been reported to be more accurate at differentiating benign from malignant polyps.^33,60,68,76,95,104^ Compliance with existing guidelines may have contributed to the increased detection of cancer above 10 mm in this meta-analysis, as findings were predominately derived from retrospective data, although the results demonstrated a clear continuous association with incremental polyp size without any significant step-change in risk at a particular threshold. Large-scale, prospective, multicentre registries are required to increase statistical power and provide better quality data to improve treatment and monitoring decisions in these patients. Randomised data would improve confidence in specific size thresholds.

There is also conflicting data regarding the cost-effectiveness of monitoring gallbladder polyps. Such analysis is dependent on accurate estimates of median cancer risk to provide meaningful analysis, which this meta-analysis can facilitate. Patel et al have suggested that compliance with polyp monitoring guidelines may be cost-effective.^81^ The authors suggested that following the European joint society guidelines^1^ would result in an estimated annual saving of £209,163 per 1000 gallbladder polyps surveyed in the National Health Service (NHS) and result in an additional 12.5% of patients requiring cholecystectomy. However, compliance with guidelines was found to be poor.^81^ Indeed, poor compliance from radiology departments is likely to represent a multifactorial problem influenced by cost, patient factors, and perceived lack of value. Given our meta-analysis demonstrates a very low risk of cancer, we suggest a health economic analysis should be conducted to evaluate the clinical value of monitoring smaller gallbladder polyps.

Strengths of our study include strict adherence to methodological and reporting recommendations, robust data extraction and quality assessment. A large volume of data from many studies and patients have been synthesised. We chose to construct the meta-analysis model in a Bayesian framework to provide greater flexibility than might be possible in a frequentist framework. As a result, we were able to develop a model that included all the studies and captured the simultaneous uncertainty that missing data, between-study heterogeneity and zero event studies bring to meta-analysis. Despite these uncertainties, the model demonstrated a clear increase in cancer risk with polyp size.

However, this study also has limitations. The analysis provides an estimate for the overall cumulative risk of cancer for different polyp sizes and the uncertainty associated with this risk. However, a clinical question not answered here is that of the conditional risk of cancer for a polyp of size greater than 10 mm, for example. This would require a far more complex model and is beyond the scope of this analysis. However, for the same reasons given in the above analysis, it is likely that any estimates of the conditional risk would also be shrouded with considerable uncertainty. As such, it is worthy of further research. We included historical data using older ultrasound technology because this review was designed to assess risk rather than technology evaluation and we wanted to capture as much follow-up data as possible. Whilst measurement error is likely to be present in older cohorts, we suggest a greater number of small polyps with less risk are likely to be detected incidentally using newer ultrasound technology, and thus contribute to a further reduction in overall malignant risk. The methodological quality of the included studies was generally considered low. Suboptimal reporting of duration and frequency of follow-up in many studies prevented meaningful modelling of cancer risk in the subsequent years after detection, which would have better informed guideline recommendations for duration of follow-up. Often, patient and polyp characteristics, including proportions of true *vs* pseudopolyps, were inadequately reported, meaning sensitivity analyses could not be performed to explore variations on our estimated median cancer risk statistics. We had planned to include high-risk patients with primary sclerosing cholangitis (PSC) as a co-variate, however there were insufficient data to allow this. Only eight patients from two included studies were reported.^81,105^ Many studies have investigated the risk of malignancy in PSC cohorts, but these can inflate the estimates in general populations and hence were excluded. Attempts were made to gather individual patient data. We received individual data from 558 patients, but the overall response rate was poor, so personalised prediction of which patients eventually developed gallbladder cancer could not be attempted. Potentially important clinical co-variates (including patient age, ethnicity, and sessile morphology) were also sporadically reported in many included studies, but addition of available co-variates in the model did not identify any factors of prognostic significance. Furthermore, any predictions are contingent on the accuracy of the model and whilst the parameter estimates were in the right direction, new trial data may refine or even challenge these. Finally, we found significant heterogeneity between studies which affected our overall confidence in the results of the meta-analysis. Publication bias could not be assessed due to the presence of intra- and inter-study heterogeneity.

## Conclusion

This review is the first comprehensive meta-analysis investigating the risk of malignancy in gallbladder polyps. Here, based on the data from 67,837 patients across 82 studies, a de novo Bayesian model was developed to establish the best available estimates concerning the development of cancer risk with polyp size. Malignant risk was extremely low, particularly in polyps measuring less than 10 mm. For polyps greater than 10 mm, estimates of the actual risk were hampered by recommended intervention in this group. However, a step increase of risk in polyps measuring larger than 10 mm is neither likely, nor supported, by these data. This suggests research efforts should be directed at improved stratification of this group and potentially increasing the threshold for intervention. Other clinical risk factors usually associated with gallbladder cancer were found to have limited effect on prediction after controlling for polyp size. Substantial heterogeneity was found between studies and the quality of evidence was generally considered low. Furthermore, this review was not able to establish how the risk of gallbladder cancer evolves over time, identifying an important gap in the evidence-base and where future research should be targeted.

## Supplementary Material

bjr.20220152.suppl-01
