# Supplementary material for: Risk of developing gallbladder cancer in patients with gallbladder polyps detected on transabdominal ultrasound: a systematic review and meta-analysis
Source: Br J Radiol. 2022 Jul 21;95(1137):20220152. doi: 10.1259/bjr.20220152 (PMC10996949; doi:10.1259/bjr.20220152)
Supplement: bjr.20220152.suppl-01 [file bjr.20220152.suppl-01.docx]

**Supplementary Material**

**Supplementary file 1.** Search strategies

**Supplementary file 2.** Data items recorded

**Supplementary file 3.** GRADE framework

**Supplementary file 4.** Development of model

**Supplementary file 5.** Probability matrix showing the percentage of polyps of given size with corresponding cancer risk

**Supplementary file 6.** QUIPS risk of bias assessments

**Supplementary file 7.** GRADE assessments

**Supplementary file 1.** Search strategies

**Ovid MEDLINE(R) ALL 1946 to December 04, 2020**

1. Gallbladder/

2. exp Polyps/

3. 1 and 2

4. ((gall bladder or gallbladder) adj5 polyp*).tw.

5. 3 or 4

6. limit 5 to (english language)

**Embase [OVID] 1947- present**

1. gallbladder polyp/

2. exp gallbladder/

3. exp polyp/

4. 2 and 3

5. ((gall bladder or gallbladder) adj5 polyp*).tw.

6. 1 or 4 or 5

7. limit 6 to English language

8. limit 7 to conference abstracts

9. 7 not 8

**Cochrane Library [Wiley]**

ID Search Hits

#1 MeSH descriptor: [Polyps] explode all trees

#2 MeSH descriptor: [Gallbladder] explode all trees

#3 #1 and #2

#4 (gallbladder or gall bladder):ti,ab,kw NEAR/5 (polyp*):ti,ab,kw

#5 #3 or #4

**Scopus**

( TITLE-ABS-KEY ( "gallbladder polyp*" ) ) OR ( TITLE-ABS-KEY ( "gall bladder polyp*" ) ) AND ( LIMIT-TO ( LANGUAGE , "English" ) )

**Web Of Science Core collection**

(English) AND DOCUMENT TYPES: (Article)

# 3 (#2 OR #1)

# 2 TS=("gall bladder polyp*")

# 1 TS=("gallbladder polyp*")

**CINAHL [Ebsco]**

S5 S3 OR S4

S4 TX (gallbladder or gall bladder) W5 *polyp*)

S3 S1 AND S2

S2 (MH "Polyps+")

S1 (MH "Gallbladder")

**Clinicaltrials.gov**

Gall bladder polyp* OR Gallbladder polyp* in condition

**Supplementary file 2.** Data items recorded

Data items recorded were:

a) Study details; primary author, publication year, article title, journal, study type, recruitment, dates of study, number of centres, sample size, numbers with missing data.

b) Participants; number of patients included, age, gender, ethnicity, co-morbidities (including gallstones and primary sclerosing cholangitis (PSC)).

c) Outcomes; total gallbladder cancers, number of cancers in patients with previously documented polyp size, time interval at which gallbladder cancer diagnosed after polyp diagnosis, blinding to polyp diagnosis.

d) Gallbladder polyp characteristics; size of gallbladder polyp, true polyp or pseudo-polyp, solitary or multiple, sessile or pedunculated, growth of polyp.

f) Clinical characteristics; follow-up time, number of patients that had cholecystectomy.

g) Results and interpretation; unadjusted and adjusted prognostic effect estimates, conclusions of study, risk of bias assessment.

**Supplementary file 3.** GRADE framework

Overall confidence in the evidence was assessed using the Grading of Recommendations Assessment, Development and Evaluation (GRADE) working group methodology.^1^

The certainty of evidence was initially classified as ‘High’ (very certain that the true effect lies close to that of the estimate of the effect) and appropriately downgraded to ‘Moderate’ (moderately certain in the effect estimate: the true effect is likely to be close to the estimate of the effect, but there is a possibility that it is substantially different), ‘Low’ (certainty in the effect estimate is limited: the true effect may be substantially different from the estimate of the effect), or ‘Very low’ (very little certainty in the effect estimate: The true effect is likely to be substantially different from the estimate of effect).

1. Guyatt GH, Oxman AD, Vist GE, et al. GRADE: an emerging consensus on rating quality of evidence and strength of recommendations. BMJ 2008;336(7650):924-26.

**Supplementary file 4.** Development of model

**Comparison of Models Developed During Analysis**

Using complete data, models were compared starting with the base model which included a random intercept only (model 1). In models 2-4 the polyp size was included as the only covariate where in model 3 it undergoes a Box-Cox transformation and in model 4 the coefficients are modelled as a bivariate normal distribution. The models were compared by taking the difference of the Deviance Information Criterion (DIC) where a decrease larger than 10 is considered to provide substantial support in favour of the new model. Based on this model 3 fitted the data better than models 1, 2, and 4.

**1. Adding Polyp Size Threshold to Base Models**

Model 1

Level 1 – Within study variation $c_{ij}\sim Binomial\left( n_{ij},\mu_{ij} \right); {logit(\mu}_{ij})= \alpha_{i}$

Level 2 – Between study variation $\alpha_{j}\sim Normal(\alpha,\sigma_{\alpha}^{2})$

Level 3 - Prior distributions $\alpha\sim uniform(-15,15)$ ; $\sigma_{\alpha} \sim uniform(0,5)$

Model 2

Level 1 – Within study variation $c_{ij}\sim Binomial\left( n_{ij},\mu_{ij} \right); {logit(\mu}_{ij})= \alpha_{i}+ \beta_{i}t_{ij}$

Level 2 – Between study variation $\alpha_{j}\sim Normal(\alpha,\sigma_{\alpha}^{2})$; $\beta_{j}\sim Normal(\beta,\sigma_{\beta}^{2})$

Level 3 - Prior distributions $\alpha\sim uniform\left( -15,15 \right);$ $\beta\sim uniform(-10,10)$

$\sigma_{\alpha} \sim uniform(0,5)$; $\sigma_{\beta} \sim uniform(0,5)$

Model 3

Level 1 – Within study variation $c_{ij}\sim Binomial\left( n_{ij},\mu_{ij} \right); {logit(\mu}_{ij})= \alpha_{i}+ \beta_{i}{BC(t}_{ij})$

Level 2 – Between study variation $\alpha_{j}\sim Normal(\alpha,\sigma_{\alpha}^{2})$; $\beta_{j}\sim Normal(\beta,\sigma_{\beta}^{2})$

Level 3 - Prior distributions $\alpha\sim uniform\left( -15,15 \right);$ $\beta\sim uniform(-10,10)$

$\sigma_{\alpha} \sim uniform(0,5)$; $\sigma_{\beta} \sim uniform(0,5)$

$$\lambda\sim uniform(0,5)$$

where $BC\left( x \right)=\left\{ \begin{aligned} \frac{x^{\lambda}-1}{\lambda} for \lambda\neq0 \\ \log\left( x \right)for \lambda=0 \end{aligned} \right.$

Model 4

Level 1 – Within study variation $c_{ij}\sim Binomial\left( n_{ij},\mu_{ij} \right); {logit(\mu}_{ij})= \alpha_{i}+ \beta_{i}t_{ij}$

Level 2 – Between study variation ${(\alpha}_{j},\beta_{j})\sim Bivariate(\left( \alpha,\beta\right), \sum)$;

Level 3 - Prior distributions $\alpha\sim uniform\left( -15,15 \right);$ $\beta\sim uniform(-10,10)$

$$\sum\sim Wishart\left( \left( \begin{matrix} 1 & 0 \\ 0 & 1 \end{matrix} \right),3 \right)$$

Results of models 1-4 are described in Tables S1 and S2.

**Table S1.** Comparison of Models when adding Polyp Size Threshold to Base Models.

| **Model** | $\bar{\boldsymbol{D}}$ | $\boldsymbol{pD}$ | $\boldsymbol{DIC}$ |
| --- | --- | --- | --- |
| 1 | 1449.8 | 52.4 | 1502.2 |
| 2 | 1053.1 | 77.2 | 1130.3 |
| 3 | 1021.6 | 66.5 | 1088.1 |
| 4 | 1032.7 | 89.1 | 1121.7 |

$\bar{D}$ = posterior mean deviance; $pD$= effective number of parameters; $DIC$ = Deviance information Criterion

**Table S2.** Difference in Deviance information Criterion between models 1-4.

| **Comparison** | **DIC difference** | **se** |
| --- | --- | --- |
| Model 2 – Model 1 | -371.9 | 122.9 |
| Model 3 – Model 1 | -414.1 | 120.3 |
| Model 4 – Model 1 | -380.5 | 125.5 |
| Model 3 – Model 2 | -42.2 | 13.2 |
| Model 4 – Model 2 | -8.6 | 10.2 |
| Model 3 – Model 4 | -33.6 | 20.0 |

$DIC$ = Deviance information Criterion; se = standard error

**2. Adding Clinical Co-variates to Polyp-size Model**

Covariates that have been previously identified as potentially predictive of Gall bladder cancer were then added to model 3 (where the polyp size is included after a Box-Cox transformation) to see if this improved on the fit provided by model 3. Again a difference in DIC < -10 was the cut off for adopting the new model. Adding a further covariate to model 3 did not improve the fit.

**Model 5**

**(including median age)**

Level 1 – Within study variation $c_{ij}\sim Binomial\left( n_{ij},\mu_{ij} \right); {logit(\mu}_{ij})= \alpha_{i}+ \beta_{i}BC(t_{ij})+\gamma{Age}_{j}$

Level 2 – Between study variation $\alpha_{j}\sim Normal(\alpha,\sigma_{\alpha}^{2})$; $\beta_{j}\sim Normal(\beta,\sigma_{\beta}^{2})$

Level 3 - Prior distributions $\alpha\sim uniform\left( -15,15 \right);$ $\beta\sim uniform(-10,10)$; $\gamma\sim Uniform (-5,5)$

$\sigma_{\alpha} \sim uniform(0,5)$; $\sigma_{\beta} \sim uniform(0,5)$

**Model 6**

**(including Male:Female odds (MFO))**

Level 1 – Within study variation $c_{ij}\sim Binomial\left( n_{ij},\mu_{ij} \right); {logit(\mu}_{ij})= \alpha_{i}+ \beta_{i}BC(t_{ij})+\gamma{MFO}_{j}$

Level 2 – Between study variation $\alpha_{j}\sim Normal(\alpha,\sigma_{\alpha}^{2})$; $\beta_{j}\sim Normal(\beta,\sigma_{\beta}^{2})$

Level 3 - Prior distributions $\alpha\sim uniform\left( -15,15 \right);$ $\beta\sim uniform(-10,10)$; $\gamma\sim Uniform (-5,5)$

$\sigma_{\alpha} \sim uniform(0,5)$; $\sigma_{\beta} \sim uniform(0,5)$

**Model 7**

**(including gallstones odds (GSO))**

Level 1 – Within study variation $c_{ij}\sim Binomial\left( n_{ij},\mu_{ij} \right); {logit(\mu}_{ij})= \alpha_{i}+ \beta_{i}BC(t_{ij})+\gamma{GSO}_{j}$

Level 2 – Between study variation $\alpha_{j}\sim Normal(\alpha,\sigma_{\alpha}^{2})$; $\beta_{j}\sim Normal(\beta,\sigma_{\beta}^{2})$

Level 3 - Prior distributions $\alpha\sim uniform\left( -15,15 \right);$ $\beta\sim uniform(-10,10)$; $\gamma\sim Uniform (-5,5)$

$\sigma_{\alpha} \sim uniform(0,5)$; $\sigma_{\beta} \sim uniform(0,5)$

**Model 8**

**(including symptoms odds (SympO))**

Level 1 – Within study variation $c_{ij}\sim Binomial\left( n_{ij},\mu_{ij} \right); {logit(\mu}_{ij})= \alpha_{i}+ \beta_{i}BC(t_{ij})+\gamma{SympO}_{j}$

Level 2 – Between study variation $\alpha_{j}\sim Normal(\alpha,\sigma_{\alpha}^{2})$; $\beta_{j}\sim Normal(\beta,\sigma_{\beta}^{2})$

Level 3 - Prior distributions $\alpha\sim uniform\left( -15,15 \right);$ $\beta\sim uniform(-10,10)$; $\gamma\sim Uniform (-5,5)$

$\sigma_{\alpha} \sim uniform(0,5)$; $\sigma_{\beta} \sim uniform(0,5)$

**Model 9**

**(including single:multiple polyp odds (SMO))**

Level 1 – Within study variation $c_{ij}\sim Binomial\left( n_{ij},\mu_{ij} \right); {logit(\mu}_{ij})= \alpha_{i}+ \beta_{i}{BC(t}_{ij})+\gamma{SMO}_{j}$

Level 2 – Between study variation $\alpha_{j}\sim Normal(\alpha,\sigma_{\alpha}^{2})$; $\beta_{j}\sim Normal(\beta,\sigma_{\beta}^{2})$

Level 3 - Prior distributions $\alpha\sim uniform\left( -15,15 \right);$ $\beta\sim uniform(-10,10)$; $\gamma\sim Uniform (-5,5)$

$\sigma_{\alpha} \sim uniform(0,5)$; $\sigma_{\beta} \sim uniform(0,5)$

Tables S3-S7 show the results of comparison between the respective clinical co-variate and the threshold model.

**Table S3.** Comparison of Models when adding Median Age to Threshold model**.**

| **Model** | **Model** | $\bar{\boldsymbol{D}}$ | $\boldsymbol{pD}$ | $\boldsymbol{DIC}$ |
| --- | --- | --- | --- | --- |
| Threshold model | 3 | 389.4 | 27.7 | 417.0 |
| Threshold model & median age | 5 | 389.1 | 27.6 | 416.7 |

$\bar{D}$ = posterior mean deviance; $pD$= effective number of parameters; $DIC$ = Deviance information Criterion. The difference in DIC between the threshold model including median age (model 5) and threshold model (model 3) was -0.31 (standard error 0.47).

**Table S4.** Comparison of Models when adding Gender to Threshold model**.**

| **Model** | **Model** | $\bar{\boldsymbol{D}}$ | $\boldsymbol{pD}$ | $\boldsymbol{DIC}$ |
| --- | --- | --- | --- | --- |
| Threshold model | 3 | 967.0 | 61.9 | 1028.9 |
| Threshold model & gender | 6 | 965.4 | 61.6 | 1027.0 |

$\bar{D}$ = posterior mean deviance; $pD$= effective number of parameters; $DIC$ = Deviance information Criterion. The difference in DIC between the threshold model including gender (model 6) and threshold model (model 3) was -1.86 (standard error 0.83).

**Table S5.** Comparison of Models when adding Gallstones to Threshold model**.**

| **Model** | **Model** | $\bar{\boldsymbol{D}}$ | $\boldsymbol{pD}$ | $\boldsymbol{DIC}$ |
| --- | --- | --- | --- | --- |
| Threshold model | 3 | 509.7 | 41.6 | 551.2 |
| Threshold model & gallstones | 7 | 511.0 | 39.8 | 550.8 |

$\bar{D}$ = posterior mean deviance; $pD$= effective number of parameters; $DIC$ = Deviance information Criterion. The difference in DIC between the threshold model including gallstones (model 7) and threshold model (model 3) was -0.42 (standard error 1.63).

**Table S6.** Comparison of Models when adding Symptoms to Threshold model**.**

| **Model** | **Model** | $\bar{\boldsymbol{D}}$ | $\boldsymbol{pD}$ | $\boldsymbol{DIC}$ |
| --- | --- | --- | --- | --- |
| Threshold model | 3 | 568.6 | 30.9 | 599.5 |
| Threshold model & symptoms | 8 | 568.5 | 31.1 | 599.6 |

$\bar{D}$ = posterior mean deviance; $pD$= effective number of parameters; $DIC$ = Deviance information Criterion. The difference in DIC between the threshold model including symptoms (model 8) and threshold model (model 3) was 0.073 (standard error 0.439).

**Table S7.** Comparison of Models when adding Single versus Multiple Polyps to Threshold model**.**

| **Model** | **Model** | $\bar{\boldsymbol{D}}$ | $\boldsymbol{pD}$ | $\boldsymbol{DIC}$ |
| --- | --- | --- | --- | --- |
| Threshold model | 3 | 716.1 | 49.6 | 765.7 |
| Threshold model & single vs multiple polyps | 9 | 716.2 | 48.5 | 764.7 |

$\bar{D}$ = posterior mean deviance; $pD$= effective number of parameters; $DIC$ = Deviance information Criterion. The difference in DIC between the threshold model including single versus multiple polyps (model 9) and threshold model (model 3) was -1.00 (standard error 2.50).

**3. Bayesian Meta-analysis Model with Box-Cox Transformation**

To predict the effects of the polyp size on the risk of gall bladder cancer Model 3 was used as the final Bayesian model that was used to calculate the results of the meta-analysis. In addition, missing data on the number of polyps and cancers at a particular size were modelled as two independent multinomial distributions based on the complete records. The two empirical distributions of the multinomial probabilities for the polyps and cancers were derived from 100 000 iterations. Each distribution consisted of a matrix of 100 000 row vectors of the multinomial probabilities. Imputation of the missing values was carried out by first uniformly sampling from each of the matrices (when appropriate) a row vector of multinomial probabilities and use these to simulate the numbers missing in each category. This process was executed for each iteration in order to establish the effects of the potential distribution of missing values on the results.

Table S8 shows the results of the parameter estimates for this Bayesian model after multiple imputation of missing data.

The model used is given below:

Level 1 – Within study variation $c_{ij}\sim Binomial\left( n_{ij},\mu_{ij} \right); {logit(\mu}_{ij})= \alpha_{i}+ \beta_{i}{BC(t}_{ij})$

Level 2 – Between study variation $\alpha_{j}\sim Normal(\alpha,\sigma_{\alpha}^{2})$; $\beta_{j}\sim Normal(\beta,\sigma_{\beta}^{2})$

Level 3 - Prior distributions $\alpha\sim uniform\left( -15,15 \right);$ $\beta\sim uniform(-10,10)$

$\sigma_{\alpha} \sim uniform(0,5)$; $\sigma_{\beta} \sim uniform(0,5)$

$$\lambda\sim uniform(0,5)$$

where $BC\left( x \right)=\left\{ \begin{aligned} \frac{x^{\lambda}-1}{\lambda} for \lambda\neq0 \\ \log\left( x \right)for \lambda=0 \end{aligned} \right.$

**Table S8.** Parameter Estimates for Bayesian Meta-analysis Model after Multiple Imputation of Missing Data.

| **Parameters** | **Mean (SD)** | **MC error** | **Median (95% CI)** |
| --- | --- | --- | --- |
| $\Lambda$ | -0.73 (0.02) | 0.002 | -0.73 (-0.77 to -0.69) |
| $\alpha$ | -14.77 (0.21) | 0.01 | -14.82 (-14.99 to -14.24) |
| $\beta$ | 8.67 (0.38) | 0.03 | 8.68 (7.90-9.38) |
| $\sigma_{\alpha}^{2}$ | 2.61 (0.98) | 0.05 | 2.46 (1.14 to 4.91) |
| $\sigma_{\beta}^{2}$ | 2.22 (0.69) | 0.02 | 2.14 (1.11 to 3.81) |

SD = Standard deviation; MC error = Monte Carlo error; CI = credible intervals.

**Supplementary file 5.** Probability matrix showing the percentage of polyps of given size with corresponding cancer risk

|  | **Polyp size** | | | | | | | | | | |
| --- | --- | --- | --- | --- | --- | --- | --- | --- | --- | --- | --- |
| **Cancer Risk** | **5 mm** | **6 mm** | **7 mm** | **8 mm** | **9 mm** | **10 mm** | **11 mm** | **12 mm** | **13 mm** | **14 mm** | **15 mm** |
| > 1 in 20 | 0% | 0% | 0% | 0% | 0% | 0% | 0% | 0% | 0% | 0% | 0% |
| > 1 in 50 | 0% | 0% | 0% | 0% | 0% | 0% | 0% | 0% | 0% | 0% | 1% |
| > 1 in 100 | 0% | 0% | 0% | 0% | 0% | 2% | 8% | 19% | 32% | 46% | 59% |
| > 1 in 250 | 0% | 1% | 16% | 54% | 83% | 94% | 98% | 99% | 100% | 100% | 100% |
| > 1 in 500 | 7% | 68% | 96% | 100% | 100% | 100% | 100% | 100% | 100% | 100% | 100% |
| > 1 in 750 | 60% | 98% | 100% | 100% | 100% | 100% | 100% | 100% | 100% | 100% | 100% |
| > 1 in 1000 | 93% | 100% | 100% | 100% | 100% | 100% | 100% | 100% | 100% | 100% | 100% |
| > 1 in 1500 | 100% | 100% | 100% | 100% | 100% | 100% | 100% | 100% | 100% | 100% | 100% |
| > 1 in 2500 | 100% | 100% | 100% | 100% | 100% | 100% | 100% | 100% | 100% | 100% | 100% |

**Supplementary file 6.** QUIPS risk of bias assessments

| **Author** | **Study Methods & Comments** | **Study Participation** | **Study Attrition** | **Prognostic Factor Measurement** | **Outcome Measurement** | **Study Confounding** | **Statistical Analysis and Reporting** | **Overall Risk of Bias** |
| --- | --- | --- | --- | --- | --- | --- | --- | --- |
| Abdullah et al^24^ | Retrospective; single-centre; patient cohort selection / baseline characteristics not adequately reported; follow-up not adequately reported; reporting quality | High | Moderate | Low | Low | Moderate | High | High |
| Ahmed et al^25^ | Retrospective; single-centre; small sample size; patient cohort selection / baseline characteristics not adequately reported; reporting quality | High | High | Low | Low | High | High | High |
| Akyurek et al^26^ | Retrospective; single-centre; small sample size; patient cohort selection / baseline characteristics not adequately reported; reporting quality | High | High | Low | Low | High | High | High |
| Al Manasra et al^27^ | Retrospective; single-centre; small sample size; patient cohort selection / baseline characteristics not adequately reported; reporting quality | High | High | Low | Low | High | High | High |
| Aldouri et al^28^ | Retrospective; single-centre; large sample size; patient cohort selection / baseline characteristics not adequately reported | Moderate | Moderate | Low | Low | Low | Moderate | Moderate |
| Aliyazicioglu et al^29^ | Retrospective; single-centre; patient cohort selection / baseline characteristics not adequately reported; reporting quality | High | High | Low | Low | High | High | High |
| Ansari et al^30^ | Prospective; single-centre; small sample size; patient cohort selection / baseline characteristics not adequately reported; follow-up not adequately reported; reporting quality | Moderate | Moderate | Low | Low | High | High | High |
| Azuma et al^31^ | Retrospective; single-centre; small sample size; patient cohort selection / baseline characteristics not adequately reported; reporting quality | High | High | Low | Low | High | High | High |
| Cairns et al^32^ | Retrospective; single-centre; large sample size; follow-up not adequately reported | Moderate | Moderate | Low | Low | Moderate | Moderate | Moderate |
| Cha et al^33^ | Retrospective; single-centre; patient cohort selection / baseline characteristics not adequately reported; reporting quality | High | High | Low | Low | Low | High | High |
| Channa et al^34^ | Retrospective; single-centre; small sample size; patient cohort selection / baseline characteristics not adequately reported; follow-up not adequately reported; reporting quality | High | High | Low | Low | High | High | High |
| Chattopadhyay et al^35^ | Retrospective; single-centre; small sample size; patient cohort selection / baseline characteristics not adequately reported; reporting quality | High | High | Low | Low | High | High | High |
| Cheon et al^36^ | Retrospective; single-centre; small sample size; patient cohort selection / baseline characteristics not adequately reported; reporting quality | HIgh | High | Low | Low | High | High | High |
| Chijiiwa et al^37^ | Retrospective; single-centre; small sample size; patient cohort selection / baseline characteristics not adequately reported; reporting quality | High | High | Low | Low | Moderate | High | High |
| Choi et al^38^ | Retrospective; single-centre; small sample size; patient cohort selection / baseline characteristics not adequately reported; follow-up not adequately reported; reporting quality | High | High | Low | Low | High | High | High |
| Chou et al^39^ | Retrospective; single-centre; large sample size; patient cohort selection / baseline characteristics not adequately reported; reporting quality | Moderate | Moderate | Low | Low | Moderate | High | High |
| Colecchia et al^40^ | Prospective; single-centre; small sample size; patient cohort selection / baseline characteristics not adequately reported; follow-up not adequately reported; reporting quality | Moderate | Moderate | Low | Low | High | High | High |
| Collett et al^41^ | Prospective; single-centre; small sample size; patient cohort selection / baseline characteristics not adequately reported | Moderate | Moderate | Low | Low | Moderate | Moderate | Moderate |
| Corwin et al^42^ | Retrospective; single-centre; patient cohort selection / baseline characteristics not adequately reported | Moderate | Moderate | Low | Low | Moderate | Moderate | Moderate |
| Csendes et al^43^ | Prospective; single-centre; patient cohort selection / baseline characteristics not adequately reported | Moderate | Moderate | Low | Low | Moderate | Moderate | Moderate |
| Dacka et al^44^ | Retrospective; single-centre; small sample size; patient cohort selection / baseline characteristics not adequately reported; reporting quality | High | High | Low | Low | High | High | High |
| Damore et al^45^ | Retrospective; single-centre; small sample size; patient cohort selection / baseline characteristics not adequately reported; reporting quality | High | High | Low | Low | High | High | High |
| Donald et al^46^ | Retrospective; single-centre; small sample size; patient cohort selection / baseline characteristics not adequately reported; follow-up not adequately reported; reporting quality | High | High | Low | Low | Moderate | High | High |
| Drews et al^47^ | Retrospective; single-centre; small sample size; patient cohort selection / baseline characteristics not adequately reported; reporting quality | High | High | Low | Low | High | High | High |
| Escalona et al^48^ | Retrospective; single-centre; patient cohort selection / baseline characteristics not adequately reported; reporting quality | High | High | Low | Low | Moderate | High | High |
| French et al^49^ | Retrospective; single-centre; patient cohort selection / baseline characteristics not adequately reported | Moderate | Moderate | Low | Low | Moderate | Moderate | Moderate |
| Fujiwara et al^50^ | Retrospective; single-centre; patient cohort selection / baseline characteristics not adequately reported; reporting quality | High | Moderate | Low | Low | Moderate | High | High |
| Guo et al^51^ | Retrospective; single-centre; patient cohort selection / baseline characteristics not adequately reported; reporting quality | High | High | Low | Low | High | High | High |
| Heitz et al^52^ | Prospective; multi-centre; small sample size; patient cohort selection / baseline characteristics not adequately reported | Low | Moderate | Low | Low | Moderate | Moderate | Moderate |
| Huang et al^53^ | Retrospective; single-centre; patient cohort selection / baseline characteristics not adequately reported; reporting quality | High | High | Low | Low | Moderate | High | High |
| Isozaki et al^54^ | Retrospective; single-centre; small sample size; patient cohort selection / baseline characteristics not adequately reported; follow-up not adequately reported; reporting quality | High | High | Low | Low | Moderate | High | High |
| Ito et al^55^ | Retrospective; single-centre; large sample size; patient cohort selection / baseline characteristics not adequately reported; follow-up not adequately reported; reporting quality | Moderate | Moderate | Low | Low | High | High | High |
| Jang et al^56^ | Prospective; single-centre; patient cohort selection / baseline characteristics not adequately reported | Moderate | Moderate | Low | Low | Moderate | Moderate | Moderate |
| Jeong et al^57^ | Retrospective; single-centre; large sample size; study design; patient cohort selection / baseline characteristics not adequately reported; reporting quality | High | High | Low | Low | High | High | High |
| Kamali Polat et al^58^ | Retrospective; single-centre; small sample size; patient cohort selection / baseline characteristics not adequately reported; follow-up not adequately reported; reporting quality | High | High | Low | Low | High | High | High |
| Khan et al^59^ | Retrospective; single-centre; small sample size; patient cohort selection / baseline characteristics not adequately reported; reporting quality | High | High | Low | Low | High | High | High |
| Kim et al^60^ | Retrospective; single-centre; small sample size; patient cohort selection / baseline characteristics not adequately reported; follow-up not adequately reported; reporting quality | High | Moderate | Low | Low | Moderate | High | High |
| Konstantinidis et al^61^ | Retrospective; single-centre; patient cohort selection / baseline characteristics not adequately reported; follow-up not adequately reported; reporting quality | High | High | Low | Low | Moderate | High | High |
| Koundouris et al^62^ | Retrospective; single-centre; small sample size; patient cohort selection / baseline characteristics not adequately reported; reporting quality | High | High | Low | Low | Moderate | High | High |
| Kratzer et al^63^ | Prospective; single-centre; small sample size; patient cohort selection / baseline characteristics not adequately reported | Moderate | Moderate | Low | Low | Moderate | Moderate | Moderate |
| Kubota et al^64^ | Retrospective; single-centre; small sample size; patient cohort selection / baseline characteristics not adequately reported; reporting quality | High | High | Low | Low | Moderate | High | High |
| Kwon et al^65^ | Retrospective; single-centre; patient cohort selection / baseline characteristics not adequately reported; follow-up not adequately reported; reporting quality | High | High | Low | Low | Low | High | High |
| Lee et al^66^ | Retrospective; single-centre; patient cohort selection / baseline characteristics not adequately reported; follow-up not adequately reported; reporting quality | High | High | Low | Low | High | High | High |
| Lee et al^67^ | Retrospective; single-centre; patient cohort selection / baseline characteristics not adequately reported; reporting quality | High | Moderate | Low | Low | Moderate | High | High |
| Liu^68^ | Retrospective; single-centre; small sample size; patient cohort selection / baseline characteristics not adequately reported; reporting quality | High | High | Low | Low | High | High | High |
| Maciejewski et al^69^ | Retrospective; single-centre; small sample size; patient cohort selection / baseline characteristics not adequately reported; reporting quality | High | High | Low | Low | High | High | High |
| Mainprize et al^70^ | Retrospective; single-centre; small sample size; patient cohort selection / baseline characteristics not adequately reported; follow-up not adequately reported; reporting quality | High | High | Low | Low | High | High | High |
| Matlok et al^71^ | Retrospective; single-centre; patient cohort selection / baseline characteristics not adequately reported; follow-up not adequately reported; reporting quality | High | High | Low | Low | High | High | High |
| Matos et al^72^ | Retrospective; single-centre; small sample size; patient cohort selection / baseline characteristics not adequately reported; follow-up not adequately reported; reporting quality | High | High | Low | Low | High | High | High |
| Metman^73^ | Retrospective; 2-centre; small sample size; patient cohort selection / baseline characteristics not adequately reported; follow-up not adequately reported; reporting quality | High | High | Low | Low | High | High | High |
| Moriguchi et al^74^ | Prospective; single-centre; patient cohort selection / baseline characteristics not adequately reported | Moderate | Moderate | Low | Low | Moderate | Moderate | Moderate |
| Okamoto et al^75^ | Very large sample size; retrospective; single-centre; patient cohort selection / baseline characteristics not adequately reported; follow-up not adequately reported | Moderate | Moderate | Low | Low | Low | Moderate | Moderate |
| Onda et al^76^ | Retrospective; single-centre; patient cohort selection / baseline characteristics not adequately reported; reporting quality | High | High | Low | Low | Low | High | High |
| Ostapenko et al^77^ | Retrospective; single-centre; small sample size; patient cohort selection / baseline characteristics not adequately reported; reporting quality | High | High | Low | Low | High | High | High |
| Park et al^78^ | Retrospective; single-centre; patient cohort selection / baseline characteristics not adequately reported; reporting quality | Moderate | High | Low | Low | Moderate | High | High |
| Park et al^79^ | Retrospective; single-centre; large sample size; patient cohort selection / baseline characteristics not adequately reported; reporting quality | High | High | Low | Low | Low | High | High |
| Park et al^80^ | Retrospective; single-centre; large sample size; patient cohort selection / baseline characteristics not adequately reported; follow-up not adequately reported; reporting quality | High | High | Low | Low | Low | High | High |
| Patel et al^81^ | Retrospective; single-centre; patient cohort selection / baseline characteristics not adequately reported; follow-up not adequately reported; reporting quality | Moderate | Moderate | Low | Low | High | High | High |
| Pedersen et al^82^ | Retrospective; single-centre; patient cohort selection / baseline characteristics not adequately reported; follow-up not adequately reported; reporting quality | Moderate | High | Low | Low | High | High | High |
| Pickering et al^83^ | Retrospective; multi-centre; small sample size; patient cohort selection / baseline characteristics not adequately reported; reporting quality | Moderate | Moderate | Low | Low | High | High | High |
| Rafaelsen et al^84^ | Prospective; single-centre; small sample size; patient cohort selection / baseline characteristics not adequately reported | Moderate | Moderate | Low | Low | Moderate | Moderate | Moderate |
| Sahiner et al^85^ | Retrospective; single-centre; small sample size; patient cohort selection / baseline characteristics not adequately reported; follow-up not adequately reported; reporting quality | High | High | Low | Low | High | High | High |
| Sarici et al^86^ | Retrospective; single-centre; small sample size; patient cohort selection / baseline characteristics not adequately reported; reporting quality | High | High | Low | Low | Low | High | High |
| Sarkut et al^87^ | Retrospective; single-centre; patient cohort selection / baseline characteristics not adequately reported; reporting quality | High | High | Low | Low | High | High | High |
| Shah^88^ | Retrospective; single-centre; small sample size; patient cohort selection / baseline characteristics not adequately reported; reporting quality | High | High | Low | Low | High | High | High |
| Shin et al^89^ | Retrospective; single-centre; patient cohort selection / baseline characteristics not adequately reported; follow-up not adequately reported; reporting quality | High | High | Low | Low | Low | High | High |
| Shinkai et al^90^ | Retrospective; single-centre; small sample size; patient cohort selection / baseline characteristics not adequately reported; follow-up not adequately reported; reporting quality | High | High | Low | Low | High | High | High |
| Spaziani et al^91^ | Retrospective; single-centre; small sample size; reporting quality | Moderate | High | Low | Low | High | High | High |
| Sugiyama et al^92^ | Retrospective; single-centre; patient cohort selection / baseline characteristics not adequately reported; follow-up not adequately reported; reporting quality | High | High | Low | Low | High | High | High |
| Sun et al^93^ | Retrospective; single-centre; patient cohort selection / baseline characteristics not adequately reported; reporting quality | High | High | Low | Low | Moderate | High | High |
| Sun et al^94^ | Retrospective; single-centre; large sample size; patient cohort selection / baseline characteristics not adequately reported; reporting quality | High | Moderate | Low | Low | Low | High | High |
| Sung et al^95^ | Retrospective; single-centre; small sample size; patient cohort selection / baseline characteristics not adequately reported; reporting quality | High | High | Low | Low | High | High | High |
| Szpakowski et al^96^ | Very large sample size; retrospective; multi-centre | Low | Moderate | Low | Low | Moderate | Moderate | Moderate |
| Terzi et al^97^ | Retrospective; single-centre; small sample size; patient cohort selection / baseline characteristics not adequately reported; reporting quality | High | High | Low | Low | Moderate | High | High |
| Terzioglu et al^98^ | Retrospective; single-centre; patient cohort selection / baseline characteristics not adequately reported; reporting quality | High | High | Low | Low | High | High | High |
| Ungarreevittaya et al^99^ | Retrospective; single-centre; small sample size; patient cohort selection / baseline characteristics not adequately reported; reporting quality | High | High | Low | Low | High | High | High |
| Velidedeoglu et al^100^ | Retrospective; single-centre; small sample size; patient cohort selection / baseline characteristics not adequately reported; reporting quality | High | High | Low | Low | High | High | High |
| Wu et al^101^ | Retrospective; single-centre; small sample size; patient cohort selection / baseline characteristics not adequately reported; reporting quality | High | High | Low | Low | High | High | High |
| Xu et al^102^ | Retrospective; single-centre; large sample size; patient cohort selection / baseline characteristics not adequately reported; follow-up not adequately reported; reporting quality | High | High | Low | Low | Moderate | High | High |
| Yang et al^103^ | Retrospective; single-centre; patient cohort selection / baseline characteristics not adequately reported; reporting quality | High | High | Low | Low | Moderate | High | High |
| Yeh^104^ | Retrospective; single-centre; patient cohort selection / baseline characteristics not adequately reported; reporting quality | High | High | Low | Low | Moderate | High | High |
| Zielinski et al^105^ | Retrospective; single-centre; patient cohort selection / baseline characteristics not adequately reported; reporting quality | High | High | Low | Low | Moderate | High | High |

Reference for QUIPS tool^1^

1. Hayden JA, van der Windt DA, Cartwright JL, et al. Assessing bias in studies of prognostic factors. Annals of internal medicine 2013;158(4):280-86.

**Supplementary file 7.** GRADE assessments

| **Author** | **GRADE** |
| --- | --- |
| Abdullah et al^24^ | Very low |
| Ahmed et al^25^ | Very low |
| Akyurek et al^26^ | Low |
| Al Manasra et al^27^ | Low |
| Aldouri et al^28^ | Moderate |
| Aliyazicioglu et al^29^ | Very low |
| Ansari et al^30^ | Low |
| Azuma et al^31^ | Low |
| Cairns et al^32^ | Moderate |
| Cha et al^33^ | Low |
| Channa et al^34^ | Very low |
| Chattopadhyay et al^35^ | Low |
| Cheon et al^36^ | Low |
| Chijiiwa et al^37^ | Low |
| Choi et al^38^ | Low |
| Chou et al^39^ | Low |
| Colecchia et al^40^ | Low |
| Collett et al^41^ | Moderate |
| Corwin et al^42^ | Moderate |
| Csendes et al^43^ | Moderate |
| Dacka et al^44^ | Very low |
| Damore et al^45^ | Very low |
| Donald et al^46^ | Low |
| Drews et al^47^ | Very low |
| Escalona et al^48^ | Low |
| French et al^49^ | Moderate |
| Fujiwara et al^50^ | Low |
| Guo et al^51^ | Low |
| Heitz et al^52^ | Moderate |
| Huang et al^53^ | Low |
| Isozaki et al^54^ | Low |
| Ito et al^55^ | Low |
| Jang et al^56^ | Moderate |
| Jeong et al^57^ | Low |
| Kamali Polat et al^58^ | Very low |
| Khan et al^59^ | Very low |
| Kim et al^60^ | Low |
| Konstantinidis et al^61^ | Low |
| Koundouris et al^62^ | Very low |
| Kratzer et al^63^ | Moderate |
| Kubota et al^64^ | Low |
| Kwon et al^65^ | Low |
| Lee et al^66^ | Low |
| Lee et al^67^ | Low |
| Liu^68^ | Low |
| Maciejewski et al^69^ | Very low |
| Mainprize et al^70^ | Low |
| Matlok et al^71^ | Low |
| Matos et al^72^ | Low |
| Metman^73^ | Low |
| Moriguchi et al^74^ | Moderate |
| Okamoto et al^75^ | Moderate |
| Onda et al^76^ | Low |
| Ostapenko et al^77^ | Low |
| Park et al^78^ | Low |
| Park et al^79^ | Low |
| Park et al^80^ | Low |
| Patel et al^81^ | Low |
| Pedersen et al^82^ | Low |
| Pickering et al^83^ | Low |
| Rafaelsen et al^84^ | Moderate |
| Sahiner et al^85^ | Low |
| Sarici et al^86^ | Low |
| Sarkut et al^87^ | Low |
| Shah^88^ | Very low |
| Shin et al^89^ | Low |
| Shinkai et al^90^ | Very low |
| Spaziani et al^91^ | Low |
| Sugiyama et al^92^ | Low |
| Sun et al^93^ | Low |
| Sun et al^94^ | Low |
| Sung et al^95^ | Low |
| Szpakowski et al^96^ | Moderate |
| Terzi et al^97^ | Low |
| Terzioglu et al^98^ | Low |
| Ungarreevittaya et al^99^ | Low |
| Velidedeoglu et al^100^ | Low |
| Wu et al^101^ | Low |
| Xu et al^102^ | Low |
| Yang et al^103^ | Low |
| Yeh^104^ | Low |
| Zielinski et al^105^ | Low |
